# Prostate Cancer, Gender Identity, and Testosterone Replacement Therapy in Klinefelter Syndrome: A Case Report and Literature Review

**DOI:** 10.7759/cureus.4630

**Published:** 2019-05-09

**Authors:** Erin Nishikawa, Sangyang Jia, Celina Dharamshi, Victoria Charron, Michael Lock

**Affiliations:** 1 Miscellaneous, Schulich School of Medicine and Dentistry, Western University, London, CAN; 2 Radiation Oncology, London Health Sciences Centre, London, CAN; 3 Radiation Oncology, Schulich School of Medicine and Dentistry, Western University, London, CAN

**Keywords:** klinefelter syndrome, prostate cancer, hypogonadism, gender identity, testosterone replacement therapy

## Abstract

Klinefelter syndrome (KS), karyotype 47, XXY, is a common cause of hypogonadism in males. Patients with this condition often experience symptoms of gonadal failure, which can precipitate gender identity challenges. Treatment with testosterone replacement therapy (TRT) can combat these symptoms by improving sexual function, muscle mass, bone health, and virilization, thereby enhancing the quality of life (QOL). Although TRT is often employed in patients with KS, there is a concern that the application of exogenous testosterone may increase the risk of prostate adenocarcinoma development and progression. We report the case of a 58-year-old male with KS who is also diagnosed with prostate adenocarcinoma and wished to remain on TRT post-radiation therapy in support of his gender identity and QOL. We describe the challenges this patient faced when balancing a rising prostate-specific antigen level and risk of cancer recurrence with his QOL.

## Introduction

Klinefelter syndrome (KS), karyotype 47, XXY, is a common cause of hypogonadism in males [1]. Patients with this condition often experience symptoms of gonadal failure, which can precipitate gender identity challenges. Treatment with testosterone replacement therapy (TRT) can combat these symptoms by improving sexual function, increasing muscle mass, protecting against osteoporosis and providing psychosexual support by maintaining virilization [1]. Although TRT is often employed in patients with KS, there is a concern that the application of exogenous testosterone may increase the risk of prostate adenocarcinoma development and progression [2]. Thus, patients with KS face a difficult risk-benefit analysis in deciding whether to use TRT. The literature contains only a few reported cases of prostate cancer in patients with KS [3-7]. We describe the case of a 58-year-old male with KS who is also diagnosed with prostate adenocarcinoma and elects to continue TRT post-radiation therapy in support of his gender identity and quality of life (QOL).

## Case presentation

A 58-year-old male with KS, diagnosed at age 17, presented to our centre. Throughout adolescence and early adulthood, he identified as female; however, he later identified as male and used TRT periodically in adulthood for two to three decades. The patient tells us the dosing and frequency of TRT during this time period was variable and intermittent; however, we were unable to obtain further details. He was diagnosed with T1 Gleason Grade 3+3 prostate adenocarcinoma six years before presenting to our centre. At the time of diagnosis, prostate-specific antigen (PSA) measured 7.79 u/L. Due to a history of traumatic experiences with the healthcare system relating to his gender identity, he had not pursued prostate cancer treatment. A second biopsy was performed after our initial consultation with the patient and revealed progression to Gleason Grade 3+4. A repeat PSA had increased marginally to 8.22 u/L. The patient had stopped TRT for much of the time period between his diagnosis of prostate cancer and presentation to our centre. Ultrasound-guided biopsy demonstrated 4/12 cores positive with 4% of tissue positive for malignancy. Clinical staging found evidence for localized disease only. Past medical history included deep vein thrombosis (DVT) following a cycling accident, hypercholesterolemia, and hypertension.

During the ensuing discussions regarding prostate cancer treatment, the patient expressed concerns regarding his symptoms of hypogonadism including weight gain and gynecomastia. Accordingly, he wished to continue TRT. He was counseled by his urologist, radiation oncologist, and endocrinologist about the possible increased risk of cancer progression in the setting of exogenous testosterone administration. He felt he was at a turning point with regard to his gender identity and had recently become sexually active, thus continuing TRT to maintain virilization was important for his QOL. Ultimately, he decided to proceed with definitive treatment in view of his cancer progression, with the caveat that he would remain on TRT. He decided upon radiation therapy rather than surgical management as this option aligned best with his values. He received local radiation 11 months after his second biopsy to a dose of 76 Gy in 35 fractions.

**Figure 1:**
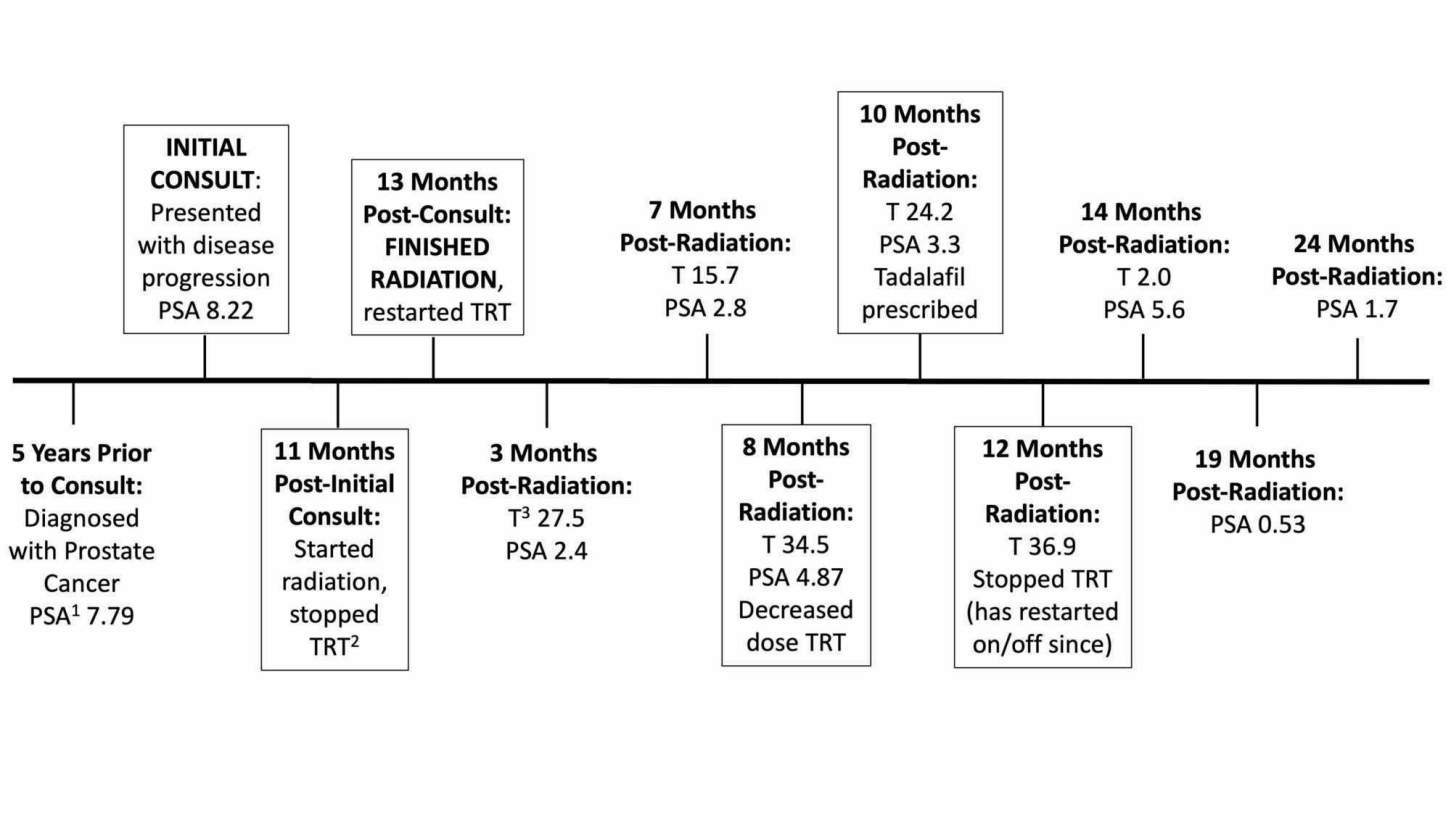
Timeline of events regarding PSA, TRT, and prostate cancer treatments PSA^1^,^ ^prostate-specific antigen in u/L; TRT^2^,^ ^testosterone replacement therapy; T^3^, testosterone in nmol/L

Post radiation therapy, a total testosterone level of 6.7-25.7 nmol/L was targeted, falling in the low-normal range for his age. Figure 1 illustrates a timeline of the patient’s diagnostic and treatment journey. Three months post radiation, his testosterone measured 27.5 nmol/L and PSA 2.4 u/L. Five months thereafter, this increased to testosterone of 34.5 nmol/L and PSA 4.87 u/L. The patient was advised that he likely has a residual disease with progression and a rapid PSA doubling time. After extensive discussion, he decreased his TRT dose by half. Around this time, he started tadalafil for erectile dysfunction and possibly due to its effectiveness, he eventually stopped TRT altogether. He attained good erectile function defined as function sufficient to complete sexual activity. At 14 months post-radiation, his testosterone dropped to 2.0 nmol/L, and PSA reached a peak of 5.6 u/L. He was advised to remain off TRT. Five months after discontinuing TRT, his PSA fell to 0.53 u/L. The responsiveness of his PSA to changes in TRT suggests that the prostate and possible residual disease remains sensitive to testosterone.

Since completing radiation therapy, the patient and his physicians have struggled to achieve a balance between adequate TRT to manage his hypogonadism symptoms and the risk of accelerating cancer progression. Long-term risk of hypogonadism includes osteoporosis; a bone mineral density showed a spine T-score of -1.4 and a femoral neck score of -0.3. After evaluating TRT risks and benefits, the patient has decided that the benefits of TRT outweigh the risk of prostate cancer progression. Under the supervision of his treatment team, the patient continues to use TRT variably four years after his initial presentation while adjusting for his PSA levels to optimize his QOL, gender identity, hypogonadism symptoms, and the risk of prostate cancer progression.

## Discussion

The implications of discontinuing TRT extend well beyond the long-term risks of hypogonadism previously described. The patient’s gender identity warrants careful consideration. Gender identity is constructed through performed gender-specific acts and expressions separate from biological sex [8]. It can be complex, with multiple identities changing within the same individual over time as occurred with our patient [9]. Importantly, a negative relationship between gender non-conformity and psychological well-being has been described [10]. Other risks associated with gender non-conformity include suicide; Bauer et al. found that among transgender individuals in Ontario, 43% had attempted suicide, with 10% attempting in the past year [11]. Thus, the decision to discontinue TRT must be weighed against the consequences of denying the patient their preferred gender representation and its possible impact on mortality.

Transgender patients may experience similar difficulties balancing gender identity and cancer risk as our patient did. A recent case report by Beswick et al. describes a female-to-male transgender patient who is diagnosed with locally advanced cervical cancer after being counseled that he did not require screening for cervical cancer because of his sexual history [12]. This patient experienced unnecessary delays in diagnosis and treatment for his cancer due to his history and gender identity, similar to our patient with KS. Healthcare providers should be cognizant of this often conflicting relationship between gender identity and cancer risk to minimize negative health outcomes for patients.

Prostatic adenocarcinoma has one of the highest cancer incidences in males, yet only five cases of this disease in patients with KS have previously been reported, with two reporting previous TRT. The first case received TRT for seven years before developing prostate adenocarcinoma and receiving radiation therapy [3]. The second was diagnosed after 35 years of TRT and underwent radical prostatectomy [4]. Notably, TRT was not re-initiated in either case. In the remaining three cases, there was no mention of TRT [5-7]. We were unable to find a case where TRT was re-initiated in a patient with KS post cancer treatment, as was our patient’s wish. However, studies do exist investigating the risks of TRT in patients without KS after treatment for prostate cancer. A retrospective study of 82 patients who received TRT after radiation therapy demonstrated minor increases in PSA [13]. This study found a low rate of prostate cancer recurrence, with biochemical recurrence occurring in approximately 6% of the patients. Others found no increased risk of cancer recurrence after TRT post-radiation therapy for prostate cancer [14-15]. A review of the literature on the use of TRT post-treatment for prostate cancer demonstrated generally no significant increases in PSA or recurrence rate [16]. However, there is a lack of randomized controlled studies or studies with large sample sizes.

Since Huggins’ study of the tumorigenic effects of exogenous androgen, attention has been placed on the potential protective effect of hypogonadism against prostatic adenocarcinoma [2]. This may explain the reduced incidence of prostate cancer in men with KS [5]. Contrary to this argument is the increased risk of prostate cancer with increasing age despite declining testosterone levels [17]. Additionally, evidence suggests that men with prostate cancer who have lower testosterone levels have higher Gleason grade disease and worse outcomes than those with normal testosterone levels [18]. It remains uncertain whether treating KS patients with TRT increases their risk of prostate adenocarcinoma.

These potential risks must be balanced with improvements in QOL for men with hypogonadism. An observational study of 999 patients with hypogonadism who received TRT demonstrated rapid and durable improvement in QOL including somatic, sexual and psychological function [19]. The studies mentioned above reported similar improvements [14-15]. Interestingly, a survey of Canadian urologists found that while the majority believed TRT was safe after prostate cancer, only a minority have actually offered TRT post-treatment [20]. Reasons for this reluctance to prescribe TRT post-cancer treatment include insufficient screening for hypogonadism. However, we must also consider a lack of understanding and weight, given to QOL concerns in comparison to the emphasis on cancer prevention.

Due to the current lack of prospective and randomized controlled trials, management of hypogonadal patients post prostate cancer treatment should be personalized to incorporate patients' values and wishes while providing education on the evidence available regarding risks and benefits of therapy. Although there is a paucity of evidence at the moment, there are no clear reasons to suggest that testosterone therapy should be a priori contraindicated in these patients. A careful follow-up with a sensitive approach to patient values and individuality will strengthen the patient-physician relationship and may lead to better functional outcomes.

## Conclusions

Our case of prostate cancer in a KS patient who wished to continue TRT after treatment highlights the importance of considering patient preferences when treating cancer. Whether TRT increases the risk for cancer recurrence or progression in this situation is inconclusive. However, there is good evidence that TRT improves QOL. An additional factor in our case is gender identity. Our patient had not sought treatment in the past, as he feared this would mean stopping TRT, thus denying him his preferred gender representation. Allowing him to continue TRT despite the risks meant that he could access treatment while maintaining his identity. This case, in addition to similar cases in the literature may provide impetus for further studies in this population enabling the development of therapeutic guidelines in the future.
